# Inhibition of KIT-Glycosylation by 2-Deoxyglucose Abrogates KIT-Signaling and Combination with ABT-263 Synergistically Induces Apoptosis in Gastrointestinal Stromal Tumor

**DOI:** 10.1371/journal.pone.0120531

**Published:** 2015-03-17

**Authors:** Thomas Mühlenberg, Susanne Grunewald, Jürgen Treckmann, Lars Podleska, Martin Schuler, Jonathan A. Fletcher, Sebastian Bauer

**Affiliations:** 1 Dept. of Medical Oncology, University Hospital Essen, University of Duisburg-Essen, Essen, Germany; 2 Sarcoma Center, University Hospital Essen, University of Duisburg-Essen, Essen, Germany; 3 Dept. of Visceral and Transplant Surgery, University Hospital Essen, University of Duisburg-Essen, Essen, Germany; 4 West German Cancer Center, University Hospital Essen, University of Duisburg-Essen, Essen, Germany; 5 German Cancer Consortium (DKTK), Heidelberg, Germany; 6 Dept. of Pathology, Brigham and Women’s Hospital, Harvard Medical School, Boston, Massachusetts, United States of America; Banner Alzheimer's Institute, UNITED STATES

## Abstract

Positron emission tomography (PET) with ^18^F-fluorodeoxyglucose (FDG) is frequently used for visualizing gastrointestinal stromal tumors (GIST), which are highly glucose-avid tumors. Dramatic metabolic responses following imatinib treatment indicate a high, KIT-dependent glucose turnover which has been particularly helpful for predicting tumor response to imatinib. The glucose analogue 2-deoxyglucose (2DG) inhibits glucose metabolism in cancer cells that depend on aerobic glycolysis for ATP production. We show that 2DG inhibits proliferation in both imatinib-sensitive and imatinib-resistant GIST cell lines at levels that can be achieved clinically. KIT-negative GIST48B have 3-14-fold higher IC50 levels than KIT-positive GIST cells indicating that oncogenic KIT may sensitize cells to 2DG. GIST sensitivity to 2DG is increased in low-glucose media (110mg/dl). 2DG leads to dose- and glucose dependent inhibition of KIT glycosylation with resultant reduction of membrane-bound KIT, inhibition of KIT-phosphorylation and inactivation of KIT-dependent signaling intermediates. In contrast to imatinib, 2DG caused ER-stress and elicited the unfolded protein response (UPR). Mannose but not pyruvate rescued GIST cells from 2DG-induced growth arrest, suggesting that loss of KIT integrity is the predominant effect of 2DG in GIST. Additive anti-tumoral effects were seen with imatinib and BH3-mimetics. Our data provide the first evidence that modulation of the glucose-metabolism by 2DG may have a disease-specific effect and may be therapeutically useful in GIST.

## Introduction

Gastrointestinal stromal tumors (GIST) are the most common mesenchymal tumors of the gastrointestinal tract and are characterized by activating mutations of the receptor tyrosine kinases KIT or PDGFRA [1,2]. Despite long-lasting responses to the tyrosine kinase inhibitor (TKI) imatinib (IM) in 80% of cases [3–5], most patients eventually progress. Second and third line therapies with the TKIs sunitinib and regorafenib rarely induce remissions but may arrest progression for a median of 5–6 months [6,7]. Patients failing all approved treatments are faced with a dismal prognosis.

Cancer cells in general have been found to commonly exhibit a high metabolic turnover. The “Warburg effect” describes a shift in glucose metabolism in cancer cells from mitochondrial respiration of pyruvate to aerobic glycolysis and production of lactic acid in the cytosol[8,9]. Compared to mitochondrial respiration, glycolysis is an inefficient mechanism of energy generation, producing two instead of 36 ATP from one glucose molecule. Given this inefficient utilization of glucose, coupled with increased energy consumption due to high proliferation rates, glucose uptake of cancer cells can be increased 200-fold over normal cells [9].

This glucose hyperconsumption is the basis for certain positron emission tomography (PET) methods, in which glucose is labeled with ^18^F (fluorodeoxyglucose, FDG) acting as a glucose analogue. Malignant tumors often show increased FDG-uptake which can be useful in distinguishing malignant from non-malignant potential in some tumors [10]. Untreated GIST are highly FDG-avid tumors that exhibit a dramatic loss of glucose uptake within 24 hours of imatinib treatment, allowing very early prediction of tumor response by PET [11].

2-deoxyglucose (2-deoxy-d-glucose, 2DG) retains the base structure of FDG but lacks the radioactive ^18^F. After being taken up by glucose transporters, 2DG is phosphorylated by hexokinase but cannot be further metabolized and thus acts as a competitive inhibitor of hexokinase, blocking metabolism of glucose to ATP. By depleting the cell of available glucose, 2DG also inhibits protein glycosylation, trapping proteins in the ER and triggering the unfolded protein response (UPR) [12,13]. As cancer cells have higher glucose demand compared to non-neoplastic tissues, disruption of glucose metabolism may be useful as therapeutic approach. Recently, 2DG has been shown to possibly inhibit MCL-1, an antiapoptotic BCL-2 protein, and the combination with ABT-263, a BH3 mimetic and BCL-2 antagonist, leads to synergistic induction of apoptosis in hematopoietic cancer models [14,15].

2DG has previously been tested alone and in combination with other cytotoxic drugs in preclinical tumor models, reducing cell viability and inducing apoptosis in lymphoma and in breast, lung and prostate cancers [14,16–18]. Recently, clinical trials have been conducted with 2DG alone or combined with chemotherapy or radiation [19,20].

Because of the high glucose uptake of GIST by FDG-PET [21] and the striking correlation of glucose-metabolism and treatment response, we hypothesize that 2DG may be particularly useful as a therapeutic strategy in GIST.

## Methods

### Reagents and Antibodies

Imatinib mesylate (IM) was purchased from Selleck Chemicals (Houston, TX). 2DG was purchased from Carbosynth (Berkshire, UK). A rabbit polyclonal antibody against KIT was from DAKO (Hamburg, Germany) and mouse monoclonal β-actin antibody was purchased from Sigma-Aldrich (Hamburg, Germany). All other antibodies used were purchased from Cell Signaling Technologies (Beverly, MA).

### Cell lines

GIST-T1 and GIST882 were established from human, untreated, metastatic GISTs and carry primary activating mutations in exons 11 and 13, respectively. GIST48 and GIST430 were established from GISTs that had progressed, after initial clinical response during IM therapy and both harbor primary activating mutations in exon 11 and secondary resistance mutations in exons 17 and 13, respectively. The exact mutations are described elsewhere[22]. GIST48B, is a subline of GIST48, which despite retaining the activating KIT mutation in all cells, expresses KIT transcript and protein at essentially undetectable levels. GIST-T1 was established by Takahiro Taguchi (Kochi University, Kochi, Japan), and all other cell lines have been established JF.

### Western Blot

Whole-cell protein lysates were prepared from cell line monolayers according to standard protocols [23]. Protein concentrations were determined with the Bio-Rad Protein Assay (Bio-Rad Laboratories, Munich, Germany). Proteins were separated by SDS/PAGE as described by Laemmli and colleagues [24] and transferred to Hybond-P membranes (GE Healthcare, Little Chalfont, UK). Changes in protein expression and phosphorylation as visualized by chemiluminescence (ECL chemi-luminescent reagent, GE Healthcare) were captured and quantified using a FUJI LAS3000 system with Science Lab 2001 ImageGauge 4.0 software (Fujifilm Medical Systems, Stamford, CT).

### Cell viability and caspase activation assays

The sulforhodamine B (SRB) assay was conducted according to the method of Skehan and colleagues [25]. Cells were plated in 96-well flat-bottomed plates. After 24 hours, culture medium was replaced with fresh medium (with or without respective drugs) in triplicate or quadruplicate cultures. At the end of drug exposure (72 hours), cells were fixed for 1 hour and stained with 0.4% SRB (Sigma Aldrich), and the optical density was detected at 560 nm. Each experiment was repeated 3 times and figures depict a representative result. For assessment of caspase 3/7 activation by Caspase Glo luminescent assay (Promega, Fitchburg, WI), cells were prepared as above and treated for 16h. Then Caspase Glo reagent was added according to manufacturer’s protocol and resulting luminescence was measured.

### Cell cycle analysis

Cells were plated in 12-well plates, grown until 80% confluence, and then treated for 24 hours with DMSO or 2DG. Cells were then trypsinized and stained with propidium iodide (Beckman Dickonson, Heidelberg, Germany) followed immediately by flow cytometric analysis (FC500 Flow Cytometer, BeckmanCoulter, Brea, CA). Modfit LT software 3.1 (Verity Software House,Topsham, ME) was used for data analysis.

### *In vivo* experiments

Animal studies were approved by "Landesamt für Natur, Umwelt und Verbraucherschutz NRW" (LANUV NRW; approval No.: 84–02.04.2012.A322). Tumor growth *in vivo* was evaluated by subcutaneously injecting the rear flanks of 6- to 8-week-old female adult athymic nude mice (NMRI nu/nu) with 1 x 10^6^ GIST-T1 cells per flank. Tumor growth was monitored biweekly with a caliper, and tumor volumes were calculated by [(length × width^2^)/2]. Mice were treated daily with 1g/kg/d orally for 14 days of 2DG dissolved in sterile H2O. After 3 weeks of treatment mice were sacrificed, and tumors were harvested. Statistical analysis of the mean tumor volumes was done by pairwise comparison using one-tailed homoscedastic t test analysis.

## Results

### 2DG has antiproliferative effects in GIST cell lines

**Fig 1:**
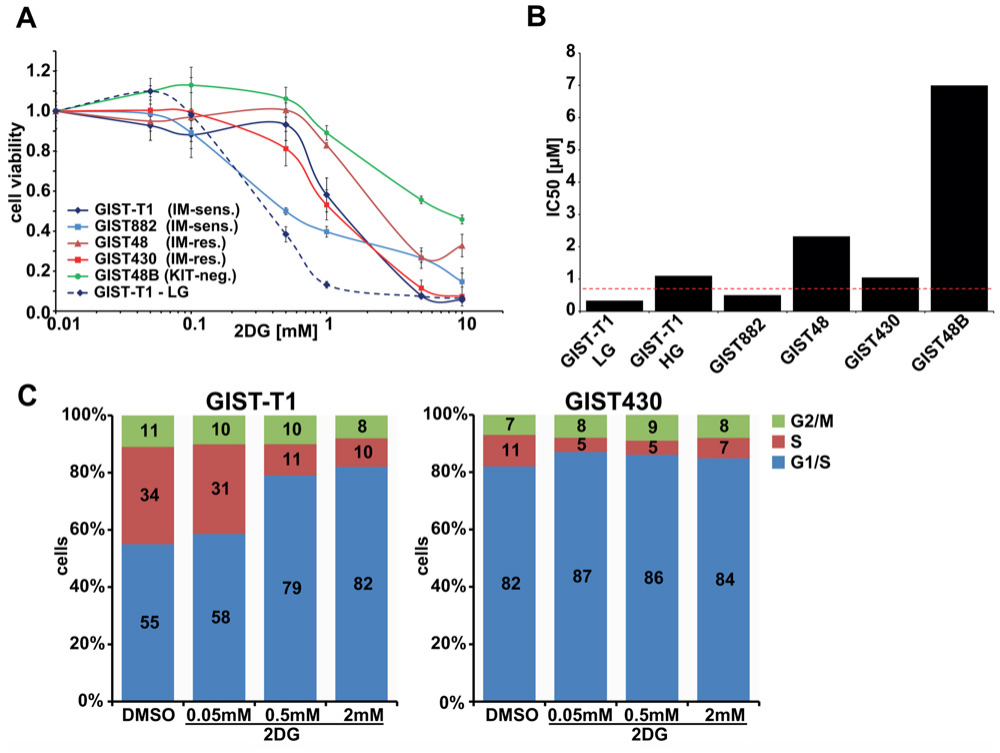
Effects of 2DG in GIST cell lines. **A**. Cytotoxicity assays (6 days) in IM-sensitive and IM-resistant GIST cell lines with increasing doses of 2DG (50μM—10mM). Dashed line represents GIST-T1 in DMEM-LG media. **B**. IC50 values depicted as bar chart with dashed line as C_max_ (0.7mM) from clinical studies (see Refs. 19–20). **C**. Cell cycle analyses after 24h treatment with increasing doses of 2DG. HG: high glucose = 4.6g/L; LG: low glucose = 1.1g/L.

Cytotoxicity assays in various IM-sensitive and IM-resistant KIT-positive GIST cell lines displayed 2DG IC50 values between 0.5μM (GIST882) and 2.5μM (GIST430; Fig. 1A). KIT-negative GIST48B was less sensitive to 2DG, with an IC50 of ~7μM (Fig. 1B). Interestingly, using media with low glucose (LG) concentration of 6mM (110mg/dL), correlating with physiological blood sugar levels, instead of 25mM (HG) led to a ~3-fold reduction of the IC50 in GIST-T1 (1.1μM to 0.33μM; Fig. 1A).

Cell cycle analyses revealed 2DG dose-dependent accumulation of cells in G1-phase and reduction of S-phase cells (Fig. 1C). This effect was more pronounced in the faster growing GIST-T1, than in GIST430.

### 2DG inhibits KIT-glycosylation in a dose- and time-dependent manner

**Fig 2:**
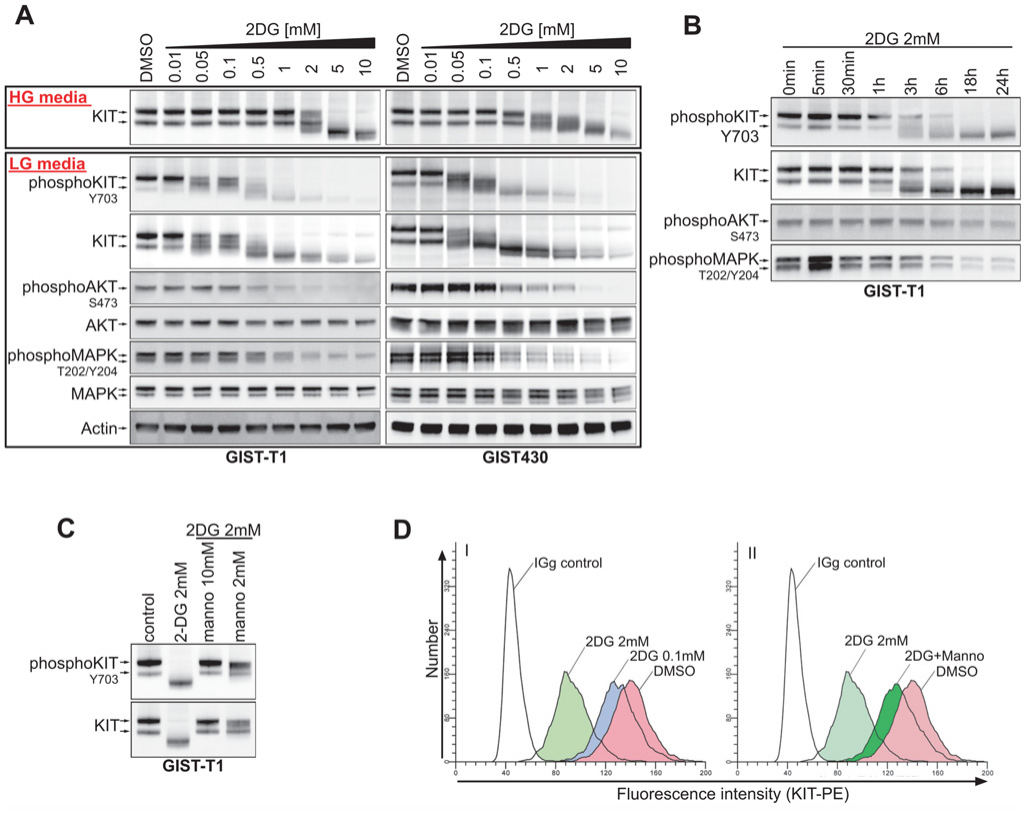
2DG inhibits KIT-glycosylation and KIT-signaling. **A**. Dose response experiments, after 24h of treatment, examining KIT-signaling in GIST cell lines growing either in HG-media (upper panel), or LG-media (lower panel). **B**. Time course experiment examining the kinetics of 2DG mediated effects in GIST-T1. **C**. Effects of co-treatment of GIST-T1 cell with 2DG and mannose on KIT-glycosylation. **D**. FACS measurement of the expression of KIT on the cell surface of GIST-T1 cells after 2DG and mannose treatment.

In 2DG dose-response studies after 24h of treatment, a KIT band shift occurred at 0.05mM for GIST-T1 cells cultured in LG-media (Fig. 2A). This reduction of protein weight raised the possibility of 2DG-mediated inhibition of KIT-glycosylation. The concentration range corresponded with a molar glucose:2DG ratio between 120:1 (at 0.05mM 2DG) and 6:1 (at 1mM 2DG). In HG-media, 2DG concentrations required to inhibit KIT-glycosylation were notably higher (2mM (**≙** 12.5:1)– 10mM (**≙** 1.5:1)). In GIST430 (HG-media) inhibition of glycosylation started at 0.5mM with complete inhibition between 5–10mM, while a reduction of glucose (LG media) showed effects at the same concentrations of 2DG as in GIST-T1 (Fig. 2A). Similar effects were observed in GIST882 (S1 Fig.). Time course studies of 2mM 2DG in GIST-T1 revealed a time-dependent reduction of KIT glycosylation reaching 50% at ~3h, in keeping with KIT-protein half-life[26] (Fig. 2B;). Adding mannose (as a glycosylation precursor) to 2DG-treated cells counteracted 2DG-effects on KIT in a dose-dependent manner; 10mM mannose maintained glycosylation at endogenous levels (Fig. 2C).

### 2DG inhibits KIT and KIT-dependent signaling

Interestingly, the observed reduction of KIT-glycosylation was accompanied by inhibition of KIT-phosphorylation. At higher concentrations (>0.1mM), inhibition of KIT-dependent AKT- and MAPK-phosphorylation ensued. However, in contrast to the reduction of KIT-protein, total expression of AKT and MAPK remained unchanged (Fig. 2A).

### 2DG treatment leads to dose-dependent change in KIT-localization

In flow cytometry (FACS) experiments, using an antibody binding to the extracellular domain of KIT, we could show that 2DG causes reduction of surface-KIT in a dose-dependent manner (Fig. 2D). Addition of 10mM mannose maintained surface-KIT expression at almost endogenous levels. The inhibition of surface-KIT expression was not antagonized by IM (S1 Fig. C).

### 2DG causes ER-Stress and elicits the unfolded protein response

**Fig 3:**
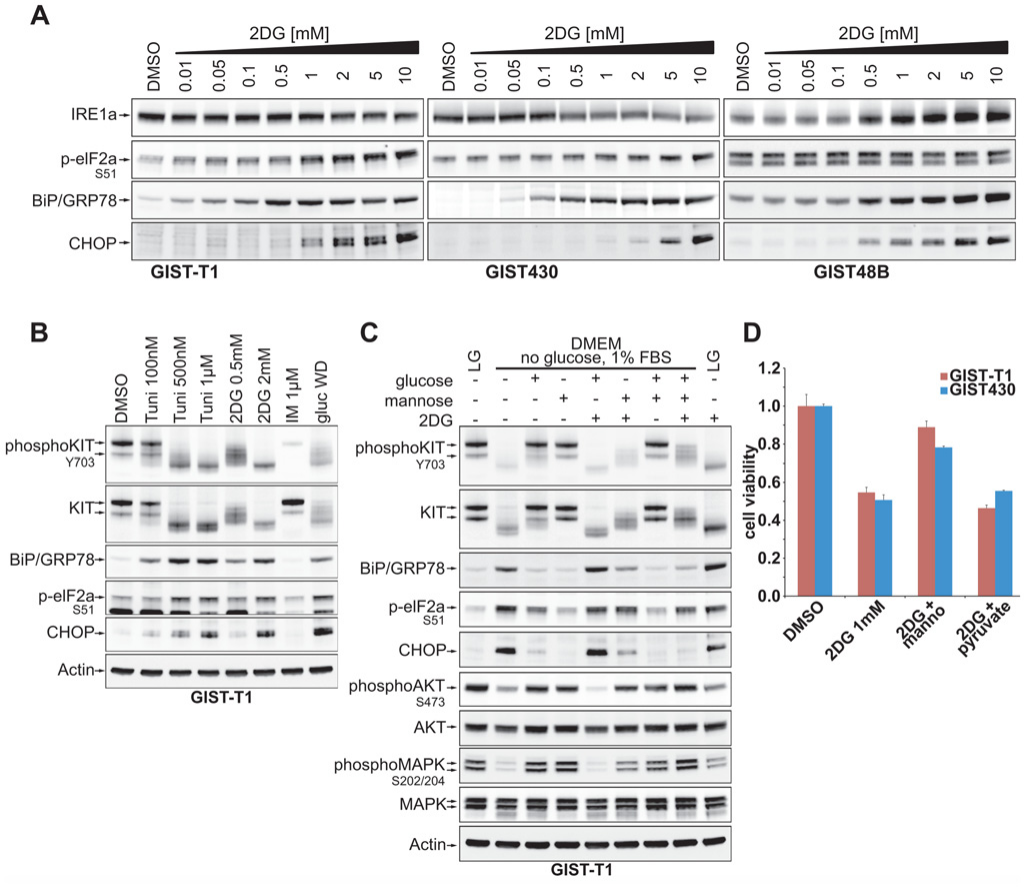
2DG mediated induction of ER-stress and the UPR A. Dose-response experiments with 2DG (0.01–10mM) show induction of markers of ER-stress and the UPR. B. Inhibition of KIT-glycosylation by tunicamycin, 2DG and withdrawal compared to inhibition of KIT-phosphorylation by IM on the induction of the UPR. C. Effects of withdrawal of Glucose and serum on GIST-T1treated with combinations of mannose and glucose (each 5mM) and 2DG. D. Rescue experiment combining 2DG with mannose and pyruvate (each 5mM).

In dose response experiments with KIT positive GIST-T1 and GIST430 and KIT-negative GIST48B we found that GRP78 (also known as BiP), a marker for ER-stress caused by unfolded proteins, was upregulated by 2DG in a dose dependent manner (Fig. 3A). In GIST430 and GIST-T1, this phenomenon was observed at 2DG concentrations 10 and 50-fold lower, respectively, than in GIST48B. Evaluations of markers for the unfolded protein response (UPR) showed that phosphorylated elF2a and its transcriptional target CHOP were induced in all of these GIST lines by 2DG, whereas IRE1a levels were induced in GIST48B but not in GIST-T1 and GIST430.

Next we sought to compare the effects of tunicamycin and 2DG with those of IM and complete glucose withdrawal. Tunicamycin is an inhibitor of N-linked glycosylation and an inducer of ER-stress and UPR. While tunicamycin and 2DG had similar effects on KIT expression and UPR, glucose withdrawal yielded a more diffuse KIT-band and a stronger induction of CHOP than the other drugs. Interestingly, imatinib did not induce ER-stress and UPR markers (Fig. 3B).

To further elucidate the effects of glucose withdrawal and 2DG on KIT-glycosylation and signaling, we starved cells for glucose and serum and added defined concentrations of glucose, mannose (each 5mM) or 2DG (2mM). As expected, complete withdrawal of glucose and serum inhibited KIT signaling and induced UPR. These effects were counteracted by glucose, and even more efficiently by mannose. Similarly, mannose was more potent than glucose in counteracting the effects of 2DG and, combined with glucose could maintain the immature form of KIT, enabling undisrupted downstream signaling (Fig. 3C).

In cytotoxicity experiments mannose counteracted the growth inhibiting effects of 2DG. On the other hand, pyruvate, the product of glycolysis which rescues cells from 2DG-induced energy starvation, did not affect cell growth (Fig. 3D).

### Combinations of 2DG with imatinib and ABT-263 enhance cytotoxic effects

**Fig 4:**
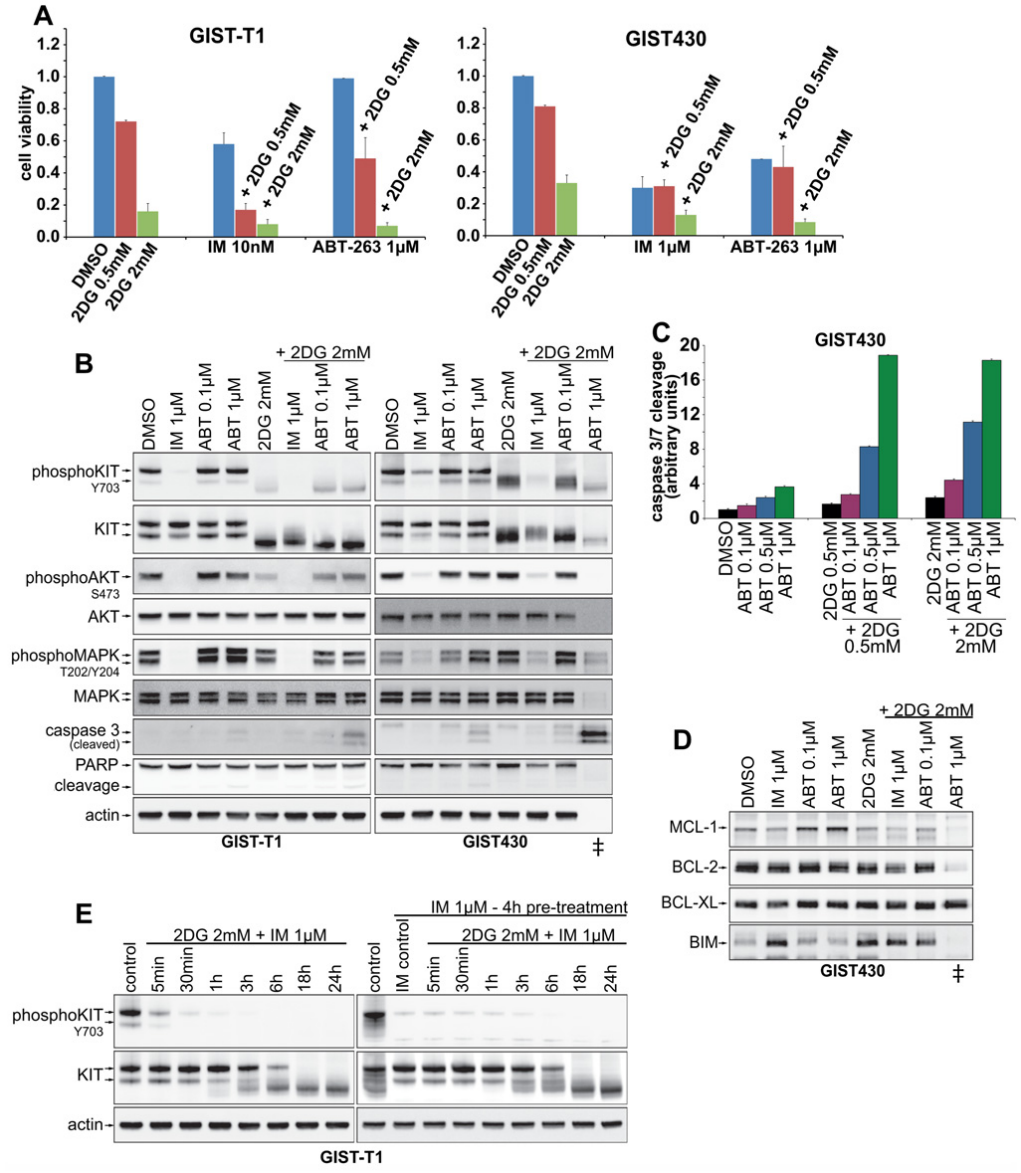
Drug combinations of 2DG with IM and ABT-263 **A**. Cytotoxicity assays (3days) combining 2DG with IM and ABT-263. **B**. Western Blot studies after 24h of co-treatment of 2DG with either IM or ABT-263. **C**. Induction of apoptosis (caspase 3/7 activation) after 16h of treatment with combinations of 2DG and ABT-263. **D**. Expression of proapoptotic (BIM) and antiapoptotic (BCL-2, BCL-XL, MCL-1) BCL-2 proteins after single and combined treatment (16h) with 2DG and the BCL-2 antagonist ABT-263.**E**. Time course studies of 2DG with IM co-treatment (left panel) and 4h IM pre-treatment before addition of 2DG (right panel).

To examine possible synergistic effects, 2DG was combined with imatinib or ABT-263. Combinations with IM had additive cytotoxic effects in IM-sensitive GIST-T1 and GIST882, while in western blots no significant induction of apoptosis, as measured by caspase 3 cleavage, was observed (Fig. 4, S1 Fig.). GIST-T1 and GIST882 displayed no response to ABT-263 1μM alone, while combination with 2DG reduced cell survival and increased cleavage of caspase 3 (Fig. 4A, B, S1 Fig.).

**Fig 5:**
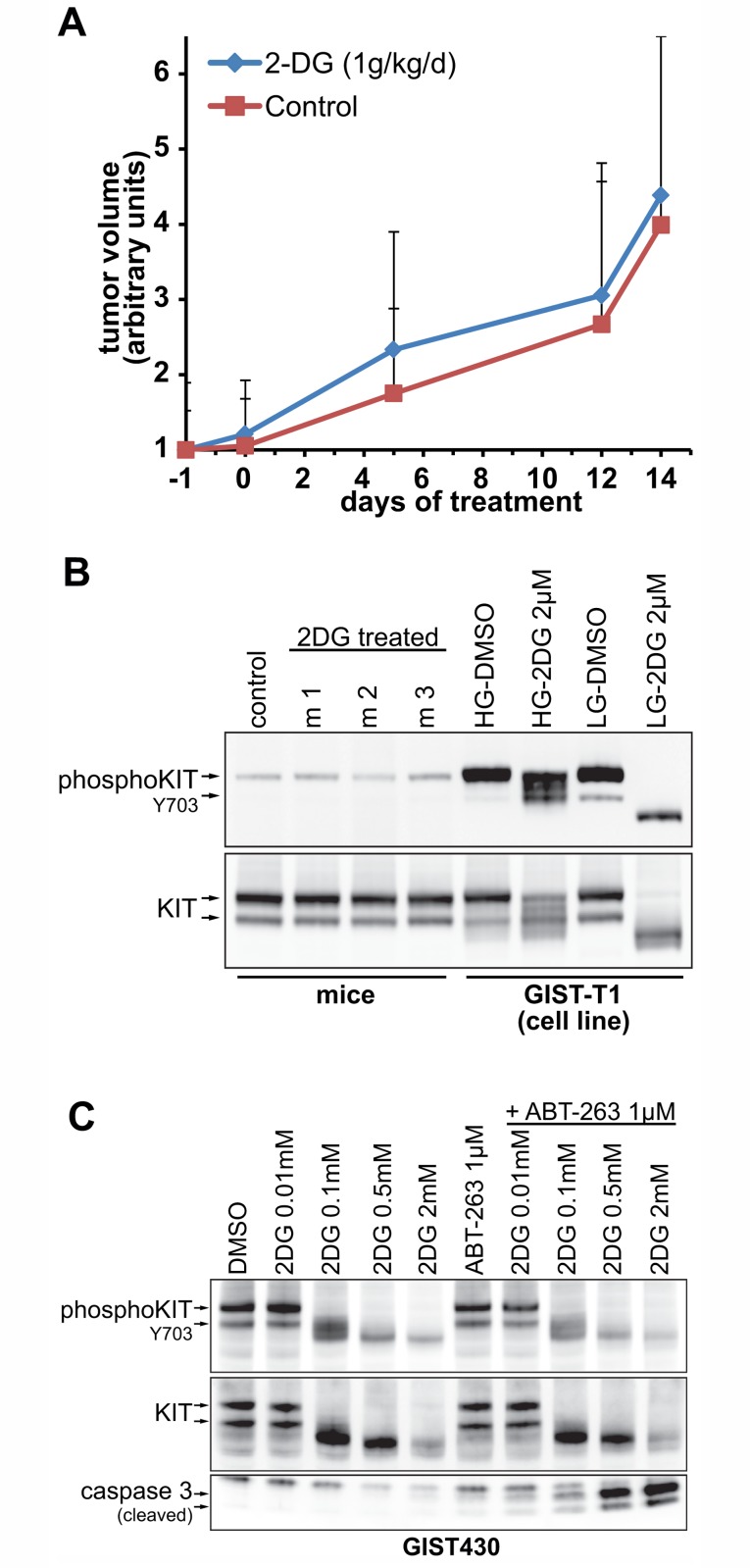
*In vivo* experiments in GIST-T1 xenografts. **A**. Growth curves of 2DG treated vs. untreated mice (normalized tumor volume in arbitrary units). **B**. Cell lysates of xenograft tumors were examined for effects of 2DG treatments on KIT and KIT-phosphorylation. **C**. Dose-Response experiment of 2DG alone or in combination with ABT-263 in GIST430 cells *in vitro*. ABT-263 only enhances apoptosis if KIT-glycosylation is inhibited by 2DG.

Strikingly, in GIST430 the combination of 2DG 2mM and ABT-263 1μM led to 92% reduction of cell viability. In 24h treatments for western blot studies (Fig. 5B), ~80% cells were detached from the dishes and therefore the cell lysates were prepared from a mix of detached and adherent cells. In these cells, apoptosis-related protein degradation had already apparently occurred, and therefore AKT, MAPK and Actin expression were not demonstrable. However, these lysates displayed very strong caspase 3 cleavage, consistent with high levels of apoptosis (Fig. 4B, lane marked with “‡”). Extending the analyses to BCL-2 family proteins, we found that 2DG slightly reduced antiapoptotic MCL-1 expression, while inducing proapoptotic BIM (Fig. 4D).

IM-treatment in patients rapidly alters GIST metabolic responses, e.g. by blocking FDG uptake. We therefore examined possible antagonistic effects of IM in co- and pre-treatments with 2DG (Fig. 4E). Interestingly, we observed no antagonistic effects in a co-treatment time course experiment with 2DG and IM given together (Fig. 4E, left panel), as compared to IM alone (Fig. 2B). When cells were pre-treated for 4h with IM, a slight delay in the onset of 2DG-mediated effects on KIT-signaling was observed after 1h-3h (Fig. 4E, right panel). However, after 18h and 24h results of co-, pre-and 2DG single agent treatment were identical (Fig. 4E, 2B).

### 2DG alone does not inhibit tumor growth in vivo

Given the promising *in vitro* data, we expanded our studies to a xenograft model of GIST-T1, being the cell line most sensitive to 2DG *in vitro*. Nude mice were treated orally with 2DG (1g/kg/d) after tumors became palpable. Surprisingly, after 2 weeks of daily treatment no inhibition of tumor growth was observed (Fig. 5A). When tumors were explanted and cell lysates were prepared, immunoblots showed no inhibition of KIT-glycosylation or KIT-phosphorylation (Fig. 5B). To test whether the previously observed apoptosis-enhancing effects of the 2DG-ABT-263 combination (Fig. 4) depended on sufficient inhibition of KIT-glycosylation, we conducted another 2DG dose-response experiment in combination with ABT-263. We found that caspase cleavage was only enhanced by the addition of ABT-263 at 2DG concentrations that disrupt KIT glycosylation (Fig. 5C).

## Discussion

GIST represents a model disease of oncogenic dependency on receptor tyrosine kinases, as evidenced by long-lasting remissions during KIT-inhibitory treatment even in patients with widely metastatic disease. Nonetheless, KIT-inhibitor treatment alone generally does not cure GIST, even among the 10–15% of patients who show ongoing response beyond 10 years of imatinib [4]. In patients who undergo resection of metastatic disease following remission to imatinib most tumor specimens contain variable amounts of viable tumor cells [27]. Both autophagy and quiescence have been shown to be mechanisms of GIST survival under imatinib treatment [28,29]. Current research aims at improving the effectiveness of KIT inhibition and combining KIT inhibition with KIT-independent targets that may improve the apoptotic treatment response. Based on clinical observations, GIST display high glucose metabolism and high expression levels of glucose transporter proteins have been shown to be associated with a poor prognosis in other sarcomas [30]. However, to date, disruption of glucose metabolism has not been investigated as a therapeutic approach in GIST.

The “Warburg effect” describes a biochemical shift of glucose metabolism commonly observed in cancer cells which utilizes aerobic glycolysis and is inefficient, requiring substantially more glucose compared to untransformed cells [8]. Warburg portrayed mitochondrial dysfunction as the cause of tumor development but for most cancers it is rather a consequence of oncogenic activation. Notably, mutations of the mitochondrial succinate dehydrogenase (SDH) have been recently found in a subset of KIT wild-type GIST [31], which may represent a confirmation of Warburg’s initial hypothesis. However, these SDH-mutant GIST are biologically distinct from KIT-mutant GIST which were studied in this work. The vast majority of GISTs high glucose turnover is dependent on oncogenic KIT activation, as the dramatic metabolic responses during imatinib treatment demonstrate [11].

Here we show that 2DG, a non-metabolizable glucose analogue, acting as an inhibitor of glucose metabolism, has cytotoxic effects in both IM-sensitive and IM-resistant GIST cell lines in the high micromolar to low millimolar range. This concentration range seems to be clinically achievable as phase 1 trials found a c_max_ of ~0.7mM in the patients’ blood after oral dosing with 2DG [19,20].

In our study 2DG concentrations as low as 50μM led to striking inhibition of the post-translational glycosylation of KIT, as evidenced by loss of the heavier (glycosylated) KIT protein form in immunoblot stains. Inhibition of KIT glycosylation was followed by inhibition of KIT phosphorylation and inhibition of KIT-dependent signaling intermediates (AKT, MAPK; Fig. 2), underscoring that post-translational modification of KIT is required for proper KIT-signaling. As previously seen with imatinib, inhibition of proliferative KIT-signaling prevents cell cycle transition with accumulation of cells in G1-phase (Fig. 1C). Similar effects on KIT-signaling were observed with tunicamycin, a specific inhibitor of N-linked glycosylation. Notably, KIT-negative GIST48B displayed the lowest sensitivity to 2DG which supports the hypothesis that oncogenic KIT may sensitize cells to 2DG. As KIT remains the oncogenic driver of GIST tumors in all stages of disease, therapies that indirectly target KIT are interesting candidates for further development.

We could show that glucose and, more potently, mannose were able to reverse the effects of 2DG on KIT which also stresses the importance of high-mannose glycosylation for KIT-signaling and thus for GIST-cell survival (Fig. 3C). In line with this, we found that in a rescue experiment mannose was indeed able to mitigate growth inhibitory effects of 2DG. Strikingly, pyruvate, the end-product of glycolysis, which should reverse 2DG induced energy deprivation, did not have beneficial effects on 2DG treated cells (Fig. 3D). These data strongly suggest that loss of KIT integrity, as mediated by inhibition of glycosylation, rather than energy deprivation by inhibition of glycolysis is the predominant effect of 2DG in GIST. This is consistent with previous observations by Kurtoglu et al. who found that under normoxic conditions 2DG, due to its similarity to mannose, mainly exerts toxicity through inhibition of N-linked glycosylation [32].

As expected, 2DG caused ER stress and elicited the unfolded protein response (UPR). To our surprise, IM did not induce ER-stress or UPR, which differs from previous findings by Nakatani et al., who demonstrated induction of GRP78 by imatinib albeit at a higher concentration of ~2μM [33]. In combined treatment, ER-stress and the UPR could be important complementary effects of 2DG, reducing TKI-induced quiescence and promoting the apoptotic response.

The combination of 2DG with IM was additive only in GIST lines with partial KIT inhibition by IM, in keeping with the supposition that KIT is the primary target of IM in these cells. These findings are also in line with our evidence that 2DG is a KIT-inhibitor in GIST and might exert additive effects only when KIT-inhibition is sub-maximal.

Against the clinical observation of imatinib-induced metabolic shut-down in imatinib-sensitive GIST we expected a substantial antagonistic impact of imatinib on 2DG-mediated effects in GIST. Tarn et al. had previously shown that expression of glucose transporters 1 and 4 (GLUT1, GLUT4) are downregulated upon IM-treatment [34]. As entry of 2DG into the cell is mediated by the same GLUTs as glucose we were surprised that 2DG effects on KIT glycosylation were slightly delayed but not inhibited by pre- or concomitant treatment with imatinib. Imatinib therefore reduces but does not fully block glucose/2DG uptake allowing for glycosylation of KIT and other glycoproteins.

Recently, 2DG was reported to inhibit the BCL-2 protein family member MCL-1, a pharmacologically challenging target, by means of a yet unknown mechanism [15]. The BCL-2 protein family consists of pro- and antiapoptotic members and minutely regulates the intrinsic pathway of apoptosis induction at the mitochondrial membrane [35]. In many cancers the equilibrium between pro- and antiapoptotic BCL-2 proteins is perturbed (e.g. by overexpression of antiapoptotic BCL-2), resulting in evasion of apoptosis [36]. Small molecules like ABT-263, so-called BH3 mimetics, antagonize antiapoptotic BCL-2 proteins, with the exception of MCL-1, for which no selective inhibitor has been developed. Importantly, in the data presented herein, combinations of 2DG with ABT-263 robustly enhanced induction of apoptosis in GIST430. We speculate that this proapoptotic activity might stem, in part, from MCL-1 inhibition by 2DG, however, additional studies will be required to address this hypothesis. In addition, we observed a BIM induction, which has been described a consequence of KIT-inhibition [37,38] but may also result from ER-stress [14] (Fig. 4).

To our surprise, GIST xenograft growth was not inhibited by 2DG. Inhibition of KIT glycosylation or phosphorylation was not detected in these 2DG-treated xenografts (Fig. 5), suggesting that typical 2DG dosing in mice is subtherapeutic from a standpoint of replicating the 2DG *in vitro* impact in GIST. This was unexpected considering that an *in vitro* glucose:2DG ratio of 120:1, which should theoretically translate into 0.001g/dL 2DG in mouse plasma, was sufficient to inhibit KIT glycosylation. We conclude that bio-availability of orally administered 2DG, at least in mice, is too low at the given dose or—alternately—that tumor cells can evade glucose starvation by alternative pathways. *In vitro* dose-response studies suggest that combination treatment with ABT-263 and 2DG yields apoptosis-enhancing effects only if the 2DG dose is sufficient to inhibit KIT-glycosylation (Fig. 5C).

In summary we find that 2DG has strong disease specific effects on GIST *in vitro* at concentrations that have been achieved in clinical trials. Apparently 2DG exerts its effects mainly through KIT-inhibition by suppressing KIT-glycosylation and the combination of 2DG with BH3-mimetics may enhance the apoptotic response. Our *in vitro* data strongly suggest therapeutic potential for 2DG if sufficient drug-levels can be achieved *in vivo*. Future studies should therefore aim to identify GIST with high 2DG sensitivity and focus on identification of alternative treatment schedules *in vivo*.

## Supporting Information

S1 Fig Effects of 2DG in GIST882 and on KIT expression A. Cytotoxicity assays (3days) combining 2DG with IM and ABT-263 in GIST882 (compare Fig. 4A). B. Western Blot studies after 24h of combination of 2DG with IM and ABT-263 in GIST882 (compare Fig. 4B). C. Expression of KIT on the cell surface of GIST-T1 cells after treatment with 2DG, IM and mannose (compare Fig. 2D). (EPS)
